# SMART Pharmacists Serving the New Needs of the Post-COVID Patients, Leaving No-One Behind

**DOI:** 10.3390/pharmacy11020061

**Published:** 2023-03-22

**Authors:** Tatjana Šipetić, Dragana Rajković, Nataša Bogavac Stanojević, Valentina Marinković, Arijana Meštrović, Michael J. Rouse

**Affiliations:** 1 AU Lilly Drogerie, Patrijarha Dimitrija 14, 11090 Belgrade, Serbia; 2 The Pharmaceutical Chamber of Serbia, Mutapova 25, 11000 Belgrade, Serbia; 3 Faculty of Pharmacy, University of Belgrade, Vojvode Stepe 450, 11000 Belgrade, Serbia; 4 Pharma Expert Consultancy and Education, Deščevec 56, 10040 Zagreb, Croatia; 5 International Services Program, Accreditation Council for Pharmacy Education (ACPE), Chicago, IL 60603, USA

**Keywords:** SMART pharmacist, community pharmacy-based intervention, post-COVID symptoms, long COVID, follow-up visit, severity of symptoms, self-medication, continuing professional development

## Abstract

This study aims to demonstrate the improvements in clinical symptoms in patients with post-COVID syndrome after a community pharmacy-based intervention in Serbia. The Pharmaceutical Chamber of Serbia (“Chamber”) invited pharmacists to deliver post-COVID patient care counselling, supported by the SMART Pharmacist Program, offering education and guidance. Present symptoms, duration and patient self-reported severity of symptoms on a scale of 1–5 on the first visit were recorded. After the counselling and proposed self-medication treatment, the time of the follow-up visit and the severity of the recorded symptoms were also recorded. The prospective data collection lasted from December 2021 to September 2022. In total, 871 patients with post-COVID symptoms were included in the study, served by 53 pharmacists. The most frequently reported post-COVID symptoms coincided with the literature, mostly related to the respiratory system (51.2%), immunity status (32.2%), fatigue and exhaustion (30.7%), skin, hair and nails (27.4%) and cognitive functions (27.9%). A total of 26.5% of patients were referred to their family physician (general practitioner), and 69.5% returned to the pharmacist for a follow-up visit. On the first visit, the median severity of patients’ symptoms was three, while on the second visit it dropped to one. The pharmacists’ intervention led to a significant improvement in the post-COVID patients’ condition.

## 1. Introduction

In the Republic of Serbia, since the first case of COVID-19 was reported on 6 March 2020, to December 2022, over 2.4 million cases of COVID-19 have been confirmed, including 17,449 deaths and a mortality rate 0.72% [1,2].

COVID-19 is a systemic disease with a wide range of clinical manifestations, ranging from asymptomatic infection to severe pneumonia, acute respiratory syndrome, acidosis, coagulation disorders, organ failure and death [3]. Older adults and patients with pre-existing chronic diseases (most commonly diabetes and cardiovascular diseases), as well as patients on immunosuppressive therapy, have a higher risk of life-threatening consequences [4].

Most people who have COVID-19 achieve full recovery within a few weeks. However, a certain number of patients, including those with mild forms of the disease, may develop post-acute COVID-19 symptoms or delayed or long-term complications of the SARS-CoV-2 infection lasting more than 4 weeks from the onset of symptoms. Various studies have been published in the past two years identifying persisting symptoms in individuals who had COVID-19, including reports from different countries across the globe. With this emerging condition, persisting symptoms linked to COVID-19 have shown to extend past the acute phase of infection, resulting in the UK’s National Institute for Health and Care Excellence (NICE) publishing a guideline for clinicians on the long-term effects of COVID-19. The guideline differentiates the terminology for long COVID and post-COVID-19 condition, formerly used interchangeably [5]. The NICE guidelines suggest the following classification, as listed in Table 1 [6]:

In addition to the clinical case definitions, the term ‘long COVID’ is commonly used to describe signs and symptoms that continue or develop after the acute phase of COVID-19. This includes both ongoing symptomatic COVID-19 (from 4 to 12 weeks) and post-COVID-19 syndrome (12 weeks or more) [6]. Based on the relapsing/remitting nature of post-COVID symptoms, Fernández-de-las-Peñas et al. proposed the integrative classification describing acute post-COVID symptoms (from week 5 to week 12), long post-COVID symptoms (from week 12 to week 24) and persistent post-COVID symptoms (lasting more than 24 weeks) [7]. In general, the pathogenesis of post-COVID syndrome remains unknown.

The Centres for Disease Control and Prevention (CDC) defined five key points for healthcare providers to consider [8]:

- “The term “post-COVID conditions” is an umbrella term for the wide range of physical and mental health consequences experienced by some patients that are present four or more weeks after SARS-CoV-2 infection, including by patients who had an initial mild or asymptomatic acute infection.
- Based on current literature, many post-COVID conditions can be managed by the primary care providers, using patient-centered approaches to optimize the quality of life of affected patients.
- Objective laboratory or imaging findings should not be used as the only measure or assessment of a patient’s well-being; as expected laboratory or imaging findings do not exclude the existence, severity, or importance of a patient’s post-COVID symptoms or conditions.
- Healthcare providers and patients are encouraged to set achievable goals through a shared decision-making patient care process, and to choose the treatment by focusing on specific symptoms (e.g., headache) or conditions (e.g., dysautonomia). A comprehensive management plan focusing on improving physical, mental, and social well-being may be helpful for some patients.
- Understanding of post-COVID conditions remains incomplete. The approach to caring for patients with post-COVID conditions will likely change over time as evidence accumulates.”

Recommendations related to care for post-COVID conditions are as follows:

After excluding serious ongoing complications or comorbidities, until the results of long-term follow-up studies are available, patients should be managed pragmatically and symptomatically with an emphasis on holistic support while avoiding over-investigation [9].

For most of the patients, the goal for the medical management of post-COVID conditions is to optimize quality of life [10].

Ideally, healthcare professionals, in consultation with relevant specialists, should develop a comprehensive management plan based on their patients’ presenting symptoms, underlying medical and psychiatric conditions, personal and social situations and their individual treatment goals [10].

As the number of COVID-19 cases and survivors grows, the burden of post-COVID-19 conditions is also expected to increase. Understanding the epidemiology and associated factors for post-COVID-19 conditions across diverse populations is crucial as the world transitions from the acute phase of the pandemic to a longer-term chronic phase [5].

The number and type of pharmaceutical services in The Republic of Serbia, at the primary level of healthcare, have been defined by the Rulebook on the Nomenclature of Health Services at the Primary Level of Healthcare since 2013. Basic services include dispensing prescription medicines and/or medicinal devices, preventive counselling on the rational and correct use of medicines and medicinal devices or medicinal–technical aids, including adherence, monitoring and reporting adverse drug reactions, and preparing compounded medications.

However, the new Law on Healthcare (2019) defines broader professional roles and responsibilities for pharmacists, especially in the field of public health, which was clearly confirmed by the adoption and implementation of the Good Pharmacy Practice (GPP) in 2021 [11].

The GPP provides standards and guidelines for the implementation of other pharmaceutical services, such as reviewing medicine use, monitoring certain parameters to optimise therapy and improving treatment outcomes (11).

The need to increase the contribution of pharmacists to healthcare systems through a greater number of services has never been more promoted than the way it has been recently. For this reason, since 2019, the Chamber started the process of the standardization of pharmaceutical services with the aim of clearly recognizing new roles, responsibilities and skills for pharmacists. Some of those services include patient care for diabetes, asthma, medicine reconciliation and antibiotic stewardship, as well as post-COVID counselling.

## 2. Materials and Methods

### 2.1. Introducing the New Community Pharmacy-Based Service to Leave No-One Behind

The need for post-COVID counselling in pharmacies was identified during the COVID-19 pandemic by the Chamber, as patients were asking for advice and help, especially for self-medication recommendations. Due to the lockdown restrictions, access to their family doctors was limited, and often even not possible. Therefore, so as to not leave anyone without pharmaceutical care services and advice, Serbian community pharmacists were called upon to deliver post-COVID patient care counselling services to upkeep social accountability and general health coverage in the country. Under the slogan “We hear you”, the strategy to leave no-one behind was clearly set by the Chamber. It was decided that this service would be organised and implemented as part of the SMART Pharmacist Program, initiated by the Accreditation Council for Pharmacy Education (ACPE) and Pharma Expert in 2014 [12]. It was designed to introduce a new continuing education model for pharmacists to support the development of competencies for future practice. SMART is an acronym for specific, measurable, achievable, relevant and timed goals for pharmacists who apply their learning into practice. In Serbia, the SMART Pharmacist Program started in 2019, under the leadership of the Chamber, to additionally extend the role of pharmacists in community pharmacies.

### 2.2. SMART Pharmacist Program—Educational Activities

In December 2021, an online educational activity for all pharmacists who registered was organised by the SMART Pharmacist Program team. The context of the educational activities focused on the newest facts and updates about COVID and post-COVID symptoms. Examples of case studies were presented to pharmacists, as well as evidence-based solutions to help patients with symptoms in the self-medication process. A standardized questionnaire was created and presented with a detailed explanation of how to approach the patient and how to fill in the questionnaire forms. As part of the SMART Pharmacist Program, the evidence-based guide for pharmacists was introduced during the educational activities and made available to all pharmacists afterwards in the form of an e-booklet. Detailed instructions for follow-up visits at pharmacies, as well as the criteria to refer patients to medical doctors, were distributed to all participants. Those guidelines advised on referral pathways, defining 3–4 weeks as the time for conducting the follow-up.

In February 2022, the second online educational activity was organized by the SMART pharmacist team, with pharmacists who had been enrolled in the previous event presenting the positive outcomes of the post-COVID counselling in pharmacies, as well as the most interesting case studies. Additionally, the newest facts and updates about post-COVID symptoms and explanations of how to use the standardized questionnaire were presented. All community pharmacists present were invited to continue or start to deliver post-COVID patient care counselling services, and to collect the results.

### 2.3. Data Collection

A cross-sectional prospective observational study was designed to identify the patients’ needs, self-reported severity of the symptoms and impact of the pharmacists’ interventions in patients with post-COVID symptoms in Serbia. The prospective data collection included the period from December 2021 to September 2022. The standardized questionnaire was developed by the lead members of the SMART Pharmacist Program, based on a review of the available literature in November 2021 [13]. There were no comprehensive published data available about the role of community pharmacy-based interventions in the improvement of clinical symptoms in patients with post-COVID syndrome; therefore, the questionnaire was based on the newest facts about symptoms of post-COVID syndrome [14,15,16].

The questionnaire consisted of two sections; the first was dedicated to the demographic data and the second to the questions about patient symptoms. The questionnaire had a list of the most common post-COVID symptoms according to the literature [2]. It included 60 symptoms targeting 14 different organ systems. For each symptom, the questionnaire had questions designed for the first and follow-up visits (Supplementary file: Questionnaire).

To document the outcomes of the first pharmacists’ intervention, there were questions about the duration of symptoms in weeks and a section for patients’ subjective descriptions of their symptom intensity and severity marked on a scale of 1–5 (where 1 stands for the lowest and 5 for the highest severity of symptoms). If there was a need to refer the patients to their family physician due to the presented symptoms, propose self-medication treatments or record the time period in weeks until the second follow-up visit, options for these were also included in the questionnaire.

For the follow-up visit, the questionnaire had questions about the patients’ self-reported severity of the recorded symptoms and use of proposed self-medication treatments. If there were any new symptoms or proposed self-medication treatment identified, those details were also included. The questionnaire included both close-ended questions with predefined answers (YES–NO) and open-ended questions. A questionnaire use pilot test was conducted by five community pharmacists for one week prior to the educational event to assess the clarity, understandability and relevance of the questionnaire items. The principal investigator held a discussion with the community pharmacists who filled out the questionnaire and, based on their feedback, checked the content validity and prepared the final version of the questionnaire. There were no significant changes other than three symptoms being added in the group “Other”.

### 2.4. Patient Counselling

Participant recruitment occurred in community pharmacies when patients approached pharmacists complaining of post-COVID symptoms or when pharmacists were asking about possible symptoms in the post-COVID period. During their conversation, the pharmacists collected subjective and objective symptoms described by the patients (pharmacist’s anamneses) when using the proposed structured algorithm in the questionnaire to learn more about patient symptoms. The inclusion criteria were based on the NICE guidelines mentioned above in the text [6]. Patients with COVID infections in the last six months confirmed by a family physician or with a COVID test were included.

The study was conducted in accordance with the Declaration of Helsinki; ethical approval for this research was obtained. The data are not publicly available as they were recorded during the provision of pharmaceutical care in community pharmacies.

Before recording the data, every patient received a brief description of the study, including that the information provided during participation in the study would remain anonymous, voluntary and confidential. The patient had to sign an informed consent to be able to participate. The pharmacists went through the whole questionnaire and asked patients about the nature of their symptoms, mentioning all the proposed clusters. If the patient confirmed having symptoms in a certain cluster, the pharmacists collected more detailed information.

During the first visit, depending on the symptoms and their severity, the pharmacist would propose self-medication treatments and suggest when the patient should come for a follow-up visit at the community pharmacy.

The protocol in the pharmacist’s booklet referenced evidence-based studies, as well as national guidelines for pharmaceutical healthcare and self-medication for pharmacists published by the Union of Pharmaceutical Associations of Serbia in collaboration with the Faculty of Pharmacy, the University of Belgrade (Guidelines for Pharmacists in Primary Healthcare, 2021), also published on the Ministry of Health government website [17].

Some of them cover indications such as diarrhoea, the application of probiotic preparations, therapy for tension-type headaches, migraine therapy, treatments of colds and the flu and acute cough therapy. These guidelines were referenced with numerous resources, such as those published by the American Pharmacists Association (OTC Advisor editions), British National Formulary, NICE guidelines, European Medicines Agency (EMA), Serbian Medicines Agency, etc.

For the nonpharmacologic interventions, were used the World Health Organization’s guide, published in 2021: Support for Rehabilitation Self-Management after COVID-19-Related Illness. Topics covered in this guide include “red flags” needing urgent attention from healthcare professionals, managing breathlessness, physical activity and exercise, energy conservation and fatigue management, managing problems with your voice, managing swallowing problems, nutrition, smell and taste problems, managing problems with attention, memory and thinking clearly, managing stress, anxiety, depression and sleep problems, managing pain, returning to work and the use of a symptom-tracking diary [16].

For all the other OTC medications, pharmacists were directed to use patient information leaflet details, and for all dietary supplements and herbal products to use allowed disease or health claims, having shown a link between a food or substance and a disease or health-related condition.

Additionally, if it was necessary due to the symptoms, the pharmacist would refer the patient to his family physician. The pharmacist would document the interventions in the questionnaire form.

During the second visit, the pharmacist would check if there was a change in the self-reported patient experience regarding the severity of the existing symptoms. The patient would be recommended to either continue with the previous self-medication treatment or to start with a new one. If it was necessary, the patient would be referred to his family physician or be scheduled for a third follow-up visit. All data about the intervention would be documented in the questionnaire form and sent by e-mail to the Chamber. Ethical approval was obtained for this study in November 2021, as described in the Institutional Review Board Statement Section. Informed consent was obtained from all patients involved in the study.

### 2.5. Statistical Analysis

Data distribution was tested using the Kolmogorov–Smirnov test. For variables with a skewed distribution, the Mann–Whitney U test and the Kruskal–Wallis test were used to analyse data differences between genders and age groups, respectively. The normally distributed variable (age) was compared between groups using Student’s *t*-test. Categorical variables were analysed with the Chi-square test. Changes in data between two repeated observations were analysed using the Wilcoxon signed-rank test. Data were shown as the mean ± standard deviation for age (normally distributed variable). Median and interquartile values were used for the presentation of independent data. Dependent data were presented as a median of difference with an interquartile range. Relative or absolute frequencies were shown for categorical variables. The statistical analyses were performed with IBM SPSS Statistics for Windows, version 27 (IBM Corp., Armonk, NY, USA). A two-tailed *p*-value ≤0.05 was considered to indicate statistical significance.

## 3. Results

All Serbian community pharmacists were invited through personal e-mail by the Chamber to participate at the first and second educational activity events. Out of the 6980 registered community pharmacists in Serbia, 441 participated at the first and 678 at the second educational activity events, but not all of them started with delivering post-COVID patient care counselling. The total number of pharmacists who offered counselling and returned questionnaires was 53. They all attended either the first, second or both educational events. In total, 51 community pharmacists became involved after the first educational activity event, and 2 new ones after the second one. Only the pharmacists who returned questionnaires were included in the study.

After the first educational activity event, the pharmaceutical Chamber’s management hosted numerous media activities, where they talked about this service with the aim of introducing it to the patients. At the same time, this media exposure influenced a large number of community pharmacists to attend the second educational event in order to learn more about this service. They saw a demonstration of the positive outcomes of post-COVID counselling provided in pharmacies by pharmacists who enrolled in the previous educational event.

Distributions of examined symptoms were evaluated in 871 patients, of which 62% were female. The mean age was 45, and males were significantly older than females. More than half of the patients had respiratory tract symptoms, and a third of them had symptoms related to cognitive functions, immunity status, fatigue and exhaustion disorders, along with skin, hair and nail changes. A similar distribution of symptoms was seen after splitting participants according to gender. Digestive system symptoms were significantly more prevalent in males, but skin, hair and nail changes were more common in females. More than one-third of study participants had one symptom, and the number of symptoms between genders was not statistically significant (*p* = 0.508). Data are shown in Table 2.

### 3.1. Analysis of the Most Frequent Symptoms According to Severity, Changes in Self-Reported Patient Symptom Severity and Pharmacist Recommendations to Patients

The self-reported severity of the most frequent symptoms at the first and the second visit and the pharmacist recommendations to patients were further analysed (Table 3). In the group of respiratory tract symptoms, the most frequent one was a cough, followed by the loss of smell and taste. The severity of the loss of taste symptom was lower than three in only 25% of patients. Almost half of the patients with chest pressure were referred to physicians. The median severity of the cognitive function, fatigue and exhaustion symptoms was three, but only a few patients were referred to physicians, while 63% of patients with symptoms related to their autoimmunity status were referred to physicians. Only one-quarter of them had symptoms at a severity of less than three. Symptoms linked to hair changes were more frequent than symptoms related to skin and nail changes. The severity was highest for the loss of hair. However, only a few patients were referred to physicians. The follow-up rate was from 64% for patients with visible changes on their skin and nails to 93% for autoimmunity disorders.

We analysed the patients’ self-reported experiences with changes in the severity of symptoms and the recommended self-medication for patients who reported the changes in the second visit. Significant changes (*p* < 0.001) were observed in the self-reported severity of the loss of smell and taste symptoms. The median difference was two, meaning that 50% of participants had a reduction in symptoms for two or more levels. The most frequently recommended self-medication in this group was alpha lipoic acid in the case of both symptoms, and olfactory retraining to recover the loss of smell (Table 4). Other self-reported symptoms significantly decreased between one and two levels in 50% of participants (*p* < 0.001). Minor changes were observed in the thickening of the skin and changes in the fingers. In total, 50% of participants were without symptom changes or had a reduced of one level. The broadest range of changes was observed for bruising, meaning that changes were not identified in 25% of patients, experiencing a reduction of two or more levels. Table 4 shows the most frequently used self-medication recommended by pharmacists at the first visit.

As explained above, the evidence-based guide for pharmacists was given to the participants to support their evidence-based counselling.

### 3.2. Analysis of the Self-Reported Symptom Severity Changes and Pharmacist Recommendations in Different Age Groups

Additionally, we compared the distribution of symptoms between age groups (Table 5). Symptoms related to impaired muscle and digestive function; changes in vital signs were significantly more prevalent in the oldest patients compared to the other two groups. The frequency of four or more symptoms was also highest in the oldest patients (*p* = 0.003).

Although the previously mentioned symptoms were different in frequency, there was no difference in severity between age groups (Table 6). The follow-up rate for all groups was similar, with the lowest being for younger patients with hearing changes (60%). For the oldest patients, the likelihood was 100% if they had symptoms of paraesthesia and impaired oxygen saturation. Regarding vital signs, most of the patients were referred to their physicians, independent of age.

**Figure 1:**
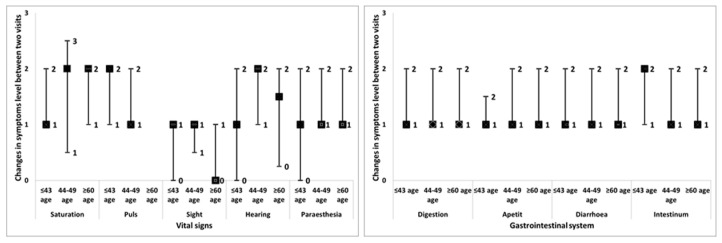
Improvement in vital signs and symptoms of the gastrointestinal system. The square represents the median value, the lower dash represents the lower quartile and the upper dash represents the upper quartile.

After the follow-up period, the reduction in gastrointestinal symptoms was significant and similar for all age groups (*p* < 0.001), as shown in Figure 1. The most frequently recommended self-medication in this group was the use of probiotics with or without prebiotics (Table 7). From the vital signs, only paraesthesia symptoms significantly improved in all groups (*p* = 0.001) in contrast with hearing symptoms, for which changes were insignificant (*p* = 0.059, *p* = 0.069 and *p* = 0.102 for the youngest, middle-aged and oldest patients, respectively). Sight and pulse symptoms improved in middle-aged patients (*p* = 0.020 and *p* = 0.004, respectively).

Additionally, pulse and oxygen saturation changed in the youngest patients (*p* = 0.007 and *p* = 0.026, respectively), and the oldest ones also had improvements in oxygen saturation (*p* = 0.026), as shown in Figure 1.

The most frequent recommendations provided by pharmacists were the use of alfa lipoic acid for reducing paraesthesia symptoms, coenzyme Q10 for pulse improvement, eye drops and eye vitamins for sight improvement and staying in fresh air for oxygen saturation symptoms (Table 7).

## 4. Discussion

### 4.1. Comparison of the Reported Symptoms with Other Studies

The systematic review and meta-analysis conducted by Lopez-Leon S. et al. aimed to identify studies assessing the long-term effects of COVID-19. It was estimated that 80% of patients infected with SARS-CoV-2 developed one or more long-term symptoms. The five most common symptoms were fatigue (58%), headaches (44%), attention disorders (27%), hair loss (25%) and dyspnoea (24%). Other symptoms concerned lung-related diseases (cough, chest discomfort, reduced pulmonary diffusing capacity, sleep apnoea and pulmonary fibrosis), cardiovascular conditions (arrhythmias and myocarditis), neurological disorders (dementia, depression, anxiety, attention disorders and obsessive–compulsive disorders) and other unspecific ones such as hair loss, tinnitus and night sweating [13]. In our research, the most common symptoms were coughing (34%), weakness and tiredness (fatigue) (27%), loss of smell (25%), loss of taste (22%), loss of energy (21%) and hair loss (19%). More recent studies obtained data from 21 articles reporting that the most common persistent clinical manifestations were fatigue (54.11%), dyspnoea (24.38%), alopecia (hair loss) (23.21%), hyperhidrosis (23.6%), insomnia (25.98%), anxiety (17.29%) and arthralgia (16.35%). In addition to these symptoms, new-onset hypertension, diabetes, neuropsychiatric disorders and bladder incontinence were also reported [18].

A couple of studies reported that fatigue was more common in females [19], and one study reported that post-activity polypnea and alopecia were more common in females [20]. This study confirmed that pattern. The rest of the studies did not align with the significance of their results by age or gender [8]. Fatigue was a common symptom in the general population, as well as in patients with acute or chronic diseases. There was no consensus on its definition or causal mechanism, but it was recognized as one of the most common symptoms of acute COVID-19, as well as post-COVID [21].

### 4.2. Changes Observed in the Patients’ Self-Reported Severity of Symptoms

The most interesting findings in this study were the significant changes observed in the patient self-reported experience of symptoms after the follow-up period, based on, but not limited to, the pharmacists’ advice and interventions. As one example, for the improvement in the loss of smell and taste symptoms, it could be concluded that alpha lipoic acid and olfactory retraining were efficient in most cases. Other similar studies had the same implications, especially evidence supporting olfactory training as a first-line intervention [22]. The role of the pharmacist in addressing these symptoms was important as most of the observed symptoms decreased between one and two levels according to the patient responses.

It is encouraging for pharmacists to embrace new roles in the area of public health education, increasing health literacy and self-medication culture in their respective communities, as many other studies confirmed [23,24].

Another interesting observation was that despite the fact that the self-reported severity of cognitive function, fatigue and exhaustion symptoms was three, only a few patients were referred to physicians. Self-medications recommended by pharmacists, such as supplements, coenzyme Q10, herbal products and nonpharmacologic interventions, such as rest, sleeping hygiene and exercise were efficient and appropriate, as patients felt better without visiting their family doctors. Fatigue was one of the earliest recognised priorities to treat in the post-COVID phase, and it is important that pharmacists leave no-one behind when these sorts of symptoms are reported at pharmacies [25].

One more example where pharmacists were found to be able to help patients either with referrals or starting self-medication counselling was the group of respiratory symptoms, especially coughing. The study results showed that more than 50% of them were successfully treated with traditional OTC antitussives, mucolytics and herbal syrups based on the pharmacists’ advice, as reported after 3 weeks of treatment. Almost half of the patients with severe symptoms were given pharmacist recommendations to visit their physicians, especially if there was no improvement in the self-medication process.

Most of the patients reporting changes in vital signs were referred to physicians independent of age. This was an important example showing how pharmacists should cooperate with other healthcare providers and base their clinical decision on their triage, especially by using standardised questionnaires and guidelines, not to miss opportunities to refer and/or conduct counselling for self-medication [26]. This was especially important during restricted access to family doctors during the pandemic, so patients did not feel neglected or left to treat themselves alone, without the advice of a healthcare provider.

Only a few patients with symptoms linked to skin and hair changes, such as the loss of hair, the thickening of the skin and changes in fingers, were referred to physicians, as, in many cases, they reported that medical doctors did not recommend any treatments. As scientific evidence does not yet point to the most successful option for patients suffering from post-COVID-19 hair loss, sometimes, medical doctors recommend only waiting for the spontaneous resolution of the disorder. The literature suggests that decisions should be determined on an individual case-by-case basis, including team-based approaches, and taking into consideration all physiological and psychosocial factors, including patient preferences [27]. As the time period for follow-up visits was very short, minor changes observed in more than 50% of participants were expected.

It was noticeable that the follow-up rate in this study was really high (from 64% for patients with visible changes for skin and nails to 93% for autoimmunity disorders), in most cases being even higher than 80%. For the oldest patients, there was a 100% chance if they had symptoms of paraesthesia and impaired oxygen saturation. It was extremely important for the successful self-medication process to have a scheduled follow-up visit with the patient to observe and record the results of the intervention. Both patient and pharmacist need to prepare for the follow-up visit, which leads to better adherence, better information exchange, open dialog and the identification of drug-related problems [28].

### 4.3. Impact of Education on Application in Practice

The SMART Pharmacist Program course for post-COVID counselling application was a need-based educational activity event. The self-assessment of pharmacist competencies was conducted as a reflection part of their learning cycle. The context of the course was precisely defined before the structure, process and outcomes of the course were defined. The methods of teaching were problem-based, encouraging the pharmacist to determine clinical decisions based on the provided guidelines and triage using a validated questionnaire. According to the globally adopted principles for pharmacy education quality, the SMART pharmacist course addressed the science, practice and ethics components of competency development [29,30]. Therefore, the clinical, economic and social impacts were expected in the long-term. Empathy, willingness to counsel and follow-up, using motivational interviewing and interventions with evidence-based OTC products were strongly recommended at the course. The reason to learn was clearer and more visible after the application of treatments and first results reported by patients. In this way, the efficacy of need-based education was confirmed, encouraging participants to be more included and apply the new knowledge, skills and attitudes in their everyday practice. Similar results were achieved in other countries, such as Turkey, Oman, Montenegro, Jordan and Qatar [28,31].

### 4.4. Limitations and Advantages of This Study

One of the limitations of this study was the use of patient-reported subjective data, describing the severity of symptoms on a scale from 1 to 5. To assure the validity of this approach, it was decided to use the principles commonly used in symptom assessments described in the literature (such as pain) [32,33]. The self-reported severity of symptoms was described as an interference with activities (disability) and the intensity of symptoms. Other aspects of the assessment could include the chronicity of the symptoms and symptom experiences (such as intensity and symptom effects).

It was not always possible to determine if the improvement in symptoms in the post-COVID period for patients was a direct result of pharmacist recommendations only or, whether it was because of any other intervention or factor, such as time. Additionally, in this study, the referrals to medical doctors between the two visits to the pharmacies were not analysed in detail, nor the influence of these visits on patient outcomes. A more detailed approach in this regard should be adopted in further investigations, after the first phase of the project. The adherence to pharmacist recommendations was not observed nor assessed. The completeness and accuracy of the laboratory data collected could not be assured, so it was decided not to include them in the results in this report.

The study did not require any external funding, as the counselling in pharmacy was a part of the regular pharmaceutical care process. The advantage of the study was the clearly defined process and data collection instructions, as well as the quality of educational activities with the follow-up to demonstrate the application of the learned knowledge and skills in practice. Patients were easily enrolled into the study, as there was a clinical need and opportunity to treat their post-COVID symptoms for the first time in pharmacies.

## 5. Conclusions

Since post-COVID symptoms affect most patients after COVID-19 infection, regardless of gender and age, pharmacists should embrace their new role in counselling patients in integrated team-based approaches to ensure no-one is left behind and to assure that any new patient needs are met. A large spectrum of post-COVID symptoms could be efficiently identified, treated and controlled in the self-medication process, based on pharmacist recommendations and counselling; however, pharmacists can also appropriately refer patients to other healthcare providers, whenever needed.

The SMART Pharmacist Program showed potential in supporting the implementation of new evidence-based services in pharmacy practices. Further investigations of community pharmacy-based interventions are needed to enhance and promote pharmacist contributions to universal health coverage in the post-COVID period.

## Figures and Tables

**Table 1:** NICE guidelines classification of COVID and post-COVID conditions [6].

Condition	Definition
Acute COVID-19	Signs and symptoms of COVID-19 present for up to 4 weeks.
Ongoing symptomatic COVID-19	Signs and symptoms of COVID-19 present from 4 weeks up to 12 weeks.
Post-COVID-19 syndrome	Signs and symptoms that develop during or after an infection consistent with COVID-19, which continue for more than 12 weeks and are not explained by an alternative diagnosis. This usually presents with clusters of symptoms, often overlapping, which can fluctuate and change over time and can affect any system in the body. Post-COVID-19 syndrome may be considered before 12 weeks while the possibility of an alternative underlying disease is also being assessed.

**Table 2:** Distributions of symptoms at the first visit in all patients, female and male.

Symptoms	All N (%)	Females N (%)	Males N (%)	*p*
N	871 (100)	534 (62)	337 (38)	
Age, y *	45 ± 15 (8–92)	44 ± 15 (8–92)	47 ± 15 (12–87)	0.008
Number of symptoms				
1	300 (34.4)	178 (33.3)	121 (36.1)	0.508
2	170 (19.5)	107 (20.0)	63 (18.6)	
3	116 (13.4)	69 (12.9)	48 (14.2)	
>3	285 (32.7)	180 (33.8)	105 (31.1)	
Respiratory system	476 (51.2)	288 (53.9)	184 (54.4)	0.888
Muscle system	222 (25.5)	131 (24.5)	90 (26.6)	0.488
Digestive system	254 (28.2)	139 (26.0)	112 (33.1)	0.024
Insomnia and sleeping disorders	170 (19.5)	110 (20.6)	59 (17.5)	0.253
Behavioural changes	121 (13.9)	76 (14.2)	35 (10.4)	0.094
Cognitive functions	243 (27.9)	152 (28.8)	89 (26.3)	0.493
Immunity status	280 (32.2)	117 (33.1)	100 (29.6)	0.273
Fatigue and exhaustion	267 (30.7)	159 (29.8)	106 (31.4)	0.620
CV system disorders	103 (11.8)	64 (12.0)	39 (11.5)	0.842
Change in vital signs	124 (14.2)	71 (13.3)	52 (15.4)	0.388
Skin, hair and nail changes	239 (27.4)	193 (36.1)	43 (12.7)	<0.001
Other	107 (12.3)	74 (13.9)	32 (9.50)	0.053

* mean ± standard deviation, (min–max). Gender groups were compared with the chi-square test (categorical data) and Student’s *t*-test (continuous data).

**Table 3:** The self-reported patient experiences of severity of symptoms at the first and second visit and the pharmacist’s referral to physicians.

The Most Frequent Symptoms	The First/Second Visit N	A Follow-Up Rate %	Referred to Physician N (%) *	The Severity of Symptoms Median (Q1–Q3) *	Symptom Severity Changes Median of Difference (Q1–Q3)
Respiratory tract					
Cough	298/252	84.6	61 (20.5)	3 (2–3)	1 (1–2)
Loss of taste	191/157	82.2	15 (7.8)	3 (2–4)	2 (1–2)
Loss of smell	216/183	84.7	20 (9.3)	3 (3–4)	2 (1–2)
Xerostomia	87/75	86.2	0 (0)	3 (2–4)	1 (1–2)
Shortness of breath	122/108	88.5	45 (36.9)	3 (2–3)	1 (1–2)
Chest pain (pressure)	119/98	82.3	58 (48.7)	3 (2–3)	1 (1–2)
Cognitive functions					
Mental tiredness	112/95	84.8	5 (4.4)	3 (2–3)	1 (1–2)
Memory, ability to remember	115/99	86.1	7 (5.0)	3 (2–3)	1 (1–2)
Ability to work, to concentrate	130/109	83.9	2 (2.2)	3 (2–3)	1 (1–2)
Daily sleepiness	90/61	67.8	9 (7.8)	3 (2–3)	1 (1–2)
Immunity status					
Frequent, repetitive infections	74/58	78.4	11 (14.9)	3 (2–3.25)	1 (1–2)
Weakness and tiredness	232/184	79.3	20 (8.5)	3 (2–3)	1 (1–2)
Autoimmune disease status	16/15	93.8	10 (62.5)	3 (3–3.75)	1 (1–2)
Fatigue and exhaustion					
Headaches	115/97	84.3	12 (10.5)	3 (2–3)	1 (1–2)
Poor physical strength	154/113	73.4	11 (7.2)	3 (2–3)	1 (1–2)
Loss of energy	182/135	74.2	9 (4.9)	3 (2–3)	1 (1–2)
Skin, hair and nail changes					
Painful scalp	27/20	74.1	0 (0)	3 (3–4)	1 (1–2)
Hair loss	163/130	81.3	6 (3.7)	4 (3–4)	1 (1–2)
Low hair quality and vitality	109/80	73.4	5 (4.6)	3 (3–4)	1 (1–2)
Dehydrated skin	93/83	89.2	2 (1.8)	3 (2–3)	1 (1–2)
Bruises	12/10	83.3	1 (8.3)	3 (2–3.75)	1 (0–2)
Thick skin	17/14	82.4	1 (5.9)	2 (1–3.5)	1 (0–1)
Visible changes on skin and nails	25/16	64.0	1 (4.0)	3 (2–3)	1 (0.25–1)

* At the first visit to the pharmacist; Q1—lower quartile (25th percentile); Q3—upper quartile (75th percentile). Changes in self-reported patient symptom severity were analysed with the Wilcoxon signed-rank test; all changes were statistically significant, *p* < 0.001.

**Table 4:** Most frequently used self-medication and other recommendations by pharmacists at the first visit.

Symptoms	N	Recommended Self-Medication 1	Recommended Self-Medication 2	Recommended Self-Medication 3	Other Recommendations
Cough	254	Acetylcysteine 25.6%	Nonprescription antitussives 13.8%	Marshmallow root extract syrup 13.0%	Herbal syrups
Loss of taste	149	Alpha lipoic acid 52.0%	Supplements (Vit A, D, B and Zn) 13.7%	Different kinds of throat lozenges 12.3%	Olfactory retraining
Loss of smell	167	Alpha lipoic acid 41.9%	Olfactory retraining 31.2%	AD drops with panthenol 13.2%	Different kinds of throat lozenges, vitamins and omega 3
Xerostomia	62	Different kinds of throat lozenges 48.4%	Nonpharmacologic interventions 21%	Mouthwash 12.9%	
Shortness of breath	80	Nonpharmacologic interventions 73.8%	Coenzyme Q10 7.5%	Inhalations 7.5%	Omega 3
Chest pain (pressure)	77	Nonpharmacologic interventions 51.9%	Coenzyme Q10 19.5%	Magnesium 10.4%	OTC analgesics and inhalations
Mental tiredness	93	Supplements (B, D, E and omega acids) 30.1%	Nonpharmacologic interventions 25.8%	Ginkgo biloba 12.9%	Supplements
Memory, ability to remember	88	Ginkgo biloba 40.1%	Supplements 29.5%	Nonpharmacologic interventions 13.6%	Rhodiola rosea
Ability to work, to concentrate	91	Omega 3 and vitamin supplements 29.6%	Ginkgo biloba 22%	Nonpharmacologic interventions 22%	Herbal supplements
Daily sleepiness	54	Nonpharmacologic interventions 33.3%	Taurine, caffeine and B12 vitamin 14.8%	Iron supplements 11.1%	Other supplements
Frequent, repetitive infections	72	Supplements (vit B, D, C, Zn, selenium and Mg) 34.4%	Acyclovir 21.3%	Beta glucan 16.4%	
Weakness and tiredness	188	Supplements 81%	Coenzyme Q10 13.3%	Fatty acids 5.7%	
Autoimmune disease status	13	Selenium, Zn and Mg supplements 46.0%	Nonpharmacologic interventions 15.4%		Supplements 38.6%
Headaches	103	OTC analgesics 62.1%	Magnesium 34.9%	Supplements 3%	
Poor physical strength	108	Supplements 28%	Nonpharmacologic interventions 24.3%	Vitamin C 18.7%	Herbal products
Loss of energy	122	Supplements 31.2%	Coenzyme Q10 26.2%	Guarana, Caffeine and Ginkgo 13.1%	
Painful scalp	18	Hair supplements formulas 66.7%	Local hair products (lotions and shampoos) 33%		Nonpharmacologic interventions
Hair loss	138	Hair supplements formulas 57.1%	Minoxidil lotion 24%		Local hair products (lotions and shampoos) and collagen and iron supplements
Low hair quality and vitality	72	Hair supplements formulas 45.5%	Nonpharmacologic interventions 39%		Local hair products (lotions and shampoos) and collagen
Dehydrated skin	86	Urea products 27.9%	Oil baths 15.1%	Emollients 14%	Nonpharmacologic interventions, supplements and cosmetics
Bruises	9	Heparin Na gel 55.5%	Chestnut extract gel 22.2%		Nonpharmacologic interventions
Thick skin	16	Urea products 57%			Nonpharmacologic interventions, supplements and cosmetics
Visible changes on skin and nails	19	Skin–hair–nail vitamin formulas 57.9%	Antimycotic 15.8%		Collagen, urea creams and nonpharmacologic interventions

**Table 5:** Distribution of symptoms in different age groups.

Symptoms	≤43 Age	44–59 Age	≥60 Age	*p* *
N	447 (51.3)	254 (29.2)	170 (19.5)	
Number of symptoms				
1	156 (34.9)	94 (37.0)	50 (29.4)	0.003
2	90 (20.1)	47 (18.5)	33 (19.4)	
3	67 (15.0)	32 (12.6)	15 (8.8)	
Respiratory tract	244 (54.5)	139 (54.7)	88 (51.8)	0.796
Muscle system	99 (22.1)	68 (26.8)	54 (31.8)	0.041
Digestive system	120 (26.8)	67 (26.4)	63 (37.1)	0.027
Insomnia and sleeping disorders	74 (16.6)	57 (22.4)	37 (21.8)	0.109
Behavioural changes	53 (11.9)	37 (14.6)	21 (12.4)	0.577
Cognitive functions	120 (26.8)	68 (26.8)	52 (30.6)	0.614
Immunity status	142 (31.8)	75 (29.5)	60 (35.3)	0.458
Fatigue and exhaustion	139 (31.1)	66 (26.0)	60 (35.3)	0.133
CV system disorders	42 (9.6)	39 (15.4)	21 (12.4)	0.076
Change in vital signs	42 (9.4)	41 (16.1)	40 (23.5)	<0.001
Skin, hair and nail changes and conditions	123 (27.5)	73 (28.7)	39 (22.9)	0.391
Other	49 (11.0)	31 (12.2)	26 (15.3)	0.339

* Age groups were compared with chi-square test.

**Table 6:** The self-reported severity of symptoms in different age groups and pharmacist referrals to physicians.

	The Self-Reported Severity of the Symptoms Median (Q1–Q3) ^#^			Follow-Up Rate %			Referred to Physician %		
Symptoms	≤43 Age	44–59 Age	≥60 Age	≤43 Age	44–59 Age	≥60 Age	≤43 Age	44–59 Age	≥60 Age
Muscle system									
Pain and cramps	3 (2–3)	3 (2–3)	3 (3–3)	82.8	91.2	96.3	8.0	11.8	20.4
Digestive system									
Digestion	3 (2–3)	3 (2–3)	3 (2–3)	88.6	88.0	84.2	7.2	8.0	15.0
Appetite	3 (2–3)	3 (2–3)	3 (2–3)	84.1	93.0	84.6	6.4	10	3.8
Diarrhoea	3 (2–3)	2 (2–3)	2.5 (2–4)	89.6	83.3	96.2	14.5	7.5	23.1
Intestinum (flatulence in intestines)	3 (2–3.5)	2 (2–3)	3 (2–4)	90.5	90.0	94.1	9.5	5.0	11.8
Vital signs									
Saturation	2 (2–3)	3 (2.8–4)	3 (2–3)	100	83.3	100	50.0	17.0	28.6
Pulse	3 (2–3.75)	3 (2–4)	2.5 (2–3)	75.0	78.6	75.0	66.6	57.1	75.0
Sight	3 (2.5–3.3)	2(1.8–3.3)	3 (1.5–3)	10	100	76.9	66.6	50.0	30.8
Hearing	3 (3–4)	3 (2–4)	3 (2.5–4)	60.0	100	80.0	70.0	40.0	60.0
Paraesthesia	3 (3–4)	3 (2–3)	3 (2–3)	92.9	92.9	100	28.6	20.0	5.0

^#^ Q1—lower quartile (25th percentile); Q3—upper quartile (75th percentile); age groups were compared with the Kruskal–Wallis test; all *p* > 0.05.

**Table 7:** Most frequently used self-medication for gastrointestinal system and vital signs recommended by pharmacists.

Symptom	N	Recommended Self-Medication 1	Recommended Self-Medication 2	Recommended Self-Medication 3	Other Recommendations
Digestion	86	Probiotics and/or prebiotics 43%	Herbal laxatives 19.7%	Plant fibres 10.6%	Supplements
Appetite	84	Vitamin B 69%	Regulated diet 11.9%	Probiotics 10.7%	Supplements
Diarrhoea	108	Probiotics and/or prebiotics 88.1%	Activated charcoal 10.2%		Regulated diet
Intestinum (flatulence in the intestines)	69	Probiotics and/or prebiotics 37.7%	Herbal extracts 15.9%	*Simethicone* 14.5%	Regulated diet
Saturation	16	Outdoor activities 56.3%	Oximeter and more frequent checks 18.8%	Breathing exercises and hyperbaric chamber 24.9%	
Pulse	10	Coenzyme Q10 50%	Magnesium 30%	Astaxanthin and other antioxidant formulas 20%	
Sight		Eyedrops 70%	Vitamin eye formulas 30%		
Hearing	13	Ginkgo 46%	Ear spray 31%	Supplements 23%	
Paraesthesia	50	Alpha lipoic acid 48%	B complex 26%		Massages and physical therapy

## Data Availability

Data available on request due to privacy restrictions. The data are not publicly available as they were recorded during the pharmaceutical care provision in community pharmacies.

## Supplementary Materials

The following supporting information can be downloaded at: https://www.mdpi.com/article/10.3390/pharmacy11020061/s1, Questionnaire.
