# Supplementary material for: SMART Pharmacists Serving the New Needs of the Post-COVID Patients, Leaving No-One Behind
Source: Pharmacy (Basel). 2023 Mar 22;11(2):61. doi: 10.3390/pharmacy11020061 (PMC10037608; doi:10.3390/pharmacy11020061)
Supplement: Supplementary file 1 [file pharmacy-2192234-supplementary.pdf › pharmacy-2192234-supplementary.pdf]

Questionnaire

PATIENT AGE										
PATIENT GENDER										
PATIENT INITIALS										
PATIENT INTERNAL CODE										
		Presenting symptoms at the first visit (availble labaratory findings or POC test)	Duration of symptoms in weeks	Severity of symptoms at the first visit (scale1-5, where 1 stands for lowest and 5 for highest severity of symptoms)	Is patient referred to his family physician? (answer YES or NO)	Proposed self-medication treatment at the first visit	Time period in weeks until the second follow-up visit	Severity of symptoms at the second follow-up visit (scale1-5, where 1 stands for lowest and 5 for highest severity of symptoms)	Presenting symptoms at the second follow-up visit (availble labaratory findings or POC test)?	Proposed self-medication treatment at the second follow-up visit
SYMPTOMS (YES or NO)										
Respiratory symptoms										
	Cough									
	Loss of taste									
	Loss of smell									
	Xerostomia									
	Shortness of breath									
	Chest pain (pressure)									
	(Does the condition worsen with exertion, lying down or sudden movement?)									
Muscle symptoms										
	Muscle pains and cramps									

Questionnaire

	(Was the pain in the same location during the infection?)									
Labaratory data (blood samples)*										
	Erythrocytes									
	Iron									
	HbA1C									
	Lipidogram (TGC i HLD/LDL)									
	Coagulation									
	Liver tests (AST/ALT)									
	The other									
Digestive system symptoms										
	Digestion									
	Appetite									
	Diarrhea									
	Intestinum flatulence in the intestines									
Liver characteristics										
	Transaminase									
	Fatty liver									
Insomnia and sleeping disorders										
	Falling asleep									
	Restless interrupted sleep									
	Anxiety									
	Nightmares									
Behavioural functions										
	Depression									
	Anxiety									

Questionnaire

	A panic attack									
	Neuroses									
	Post-traumatic stress disorder									
Cognitive functions										
	Mental tirredness									
	Memory, ability to remember									
	Ability to work, to concentrate									
	Daily sleepiness									
Immunity status										
	Frequent, repetitive infections									
	Weakness and tiredness									
	Autoimmune disease status									
Fatigue and exhaustion										
	Headaches									
	Poor physical strength									
	Loss of energy									
Cardiovascular system disorders										
	Arrhythmia									
	Hypertension									
	Changes in the blood count									
	Electrocardiogram, Holter monitor, Ultrasound									
Change of vital signs										
	Saturation									
	Pulse									
	Sight									

Questionnaire

	Hearing									
	Paresthesia<br>(tingling, prickling, burning, cooling)									
Skin, hair and nail changes and conditions										
	Painful scalp									
	Hair loss									
	Low hair quality and vitality									
	Dehydrated skin									
	Bruisers									
	Thick skin									
	Visible changes on skin and nails									
The Other										
	Menstruation disorders									
	Worsening of chronic diseases									
	Change in body weight									
	Difficulty swallowing									
